# Heightened Sensitivity of the Hyperexcitable Occipital Cortex to Spreading Depression: Evidence for State-Dependent Mechanisms of Migraine Aura

**DOI:** 10.3390/neurolint18050097

**Published:** 2026-05-21

**Authors:** Tatiana M. Medvedeva, Maria P. Smirnova, Lyudmila V. Vinogradova

**Affiliations:** Institute of Higher Nervous Activity and Neurophysiology, Russian Academy of Sciences, Butlerova Street 5A, 117485 Moscow, Russia; golova93tanya@gmail.com (T.M.M.); rymarik@gmail.com (M.P.S.)

**Keywords:** migraine, aura, cortical spreading depression, spreading depolarization, hyperexcitability

## Abstract

**Background/Objectives**: Cortical spreading depolarization (SD) is recognized as the pathophysiological substrate of migraine aura. Suppression of ongoing cortical activity produced by SD is thought to underlie the transient neurological deficits characteristic of the aura phase. While cortical hyperexcitability is a well-established feature of migraine brain, the effect of SD on spontaneous electrical activity in the hyperexcitable cortex remains poorly understood. Here, we investigate how SD and SD-induced depression of cortical activity are modulated by a state of mildly enhanced excitability. **Methods**: Using freely behaving rats, we assessed characteristics of SDs, electrocorticographic spectral power in the frontal and occipital cortices during interictal period and after SD initiation, under both drug-free conditions and following mild pharmacological disinhibition. **Results**: Mild cortical disinhibition resulted in a significant increase in baseline oscillatory power relative to control conditions. While cortical hyperexcitability did not alter the properties of SD itself, it differentially modulated the impact of SD on spontaneous activity in a region-specific manner. Notably, under conditions of enhanced excitability, the duration of SD-induced depression was markedly reduced in the frontal cortex but prolonged in the occipital cortex. **Conclusions**: These findings demonstrate that the effects of SD on spontaneous cortical activity are critically dependent on the baseline level of cortical excitability and exhibit distinct regional heterogeneity. In the awake, hyperexcitable state, the occipital cortex shows heightened vulnerability to SD-induced depression, a finding that may provide a mechanistic basis for the disproportionate involvement of the occipital cortex in aura generation and the predominance of visual symptoms in migraine aura.

## 1. Introduction

Migraine is a common neurological disorder affecting approximately 15% of the global population, with a high burden in individuals of working age. Migraine is characterized by recurrent attacks of unilateral headaches of moderate-to-severe intensity and sensory hypersensitivity. Migraine with aura is diagnosed in about one-third of patients who experience transient neurological disturbances before the onset of headache. The most common type of migraine aura is visual (associated with scotoma, phosphenes scintillations, etc.). The pathophysiological mechanism of migraine aura is cortical spreading depolarization (SD), a wave of massive cellular depolarization slowly propagating over the cortex and producing transient dysfunction of the affected tissue [1,2]. Reversible depression of ongoing activity is a reliable manifestation of cortical SD that presumably underlies spreading neurological deficit in migraine aura [1,2,3,4]. Propagating suppression of cortical activity during visual aura has been detected in migraine patients using fMRI, MEG and EEG approaches [5,6,7].

The cortex of patients with migraine, particularly migraine with aura and chronic migraine, is hyperexcitable [8,9,10]. It is suggested that the hyperexcitable state predisposes the cortex to triggering SD in response to extrinsic or intrinsic stimuli [8,11,12]. To date, it is unknown whether a mild increase in cortical excitability affects the characteristics of SD and SD-induced depression. Main information about properties and consequences of SD has been obtained in pre-clinical studies performed in anesthetized animals, i.e., in a cortex with reduced baseline excitability. In the hippocampi of awake rats, SD exerts distinct electrographic effects in regions with increased baseline excitability [13]. We hypothesize that cortical hyperexcitability can alter SD and its effects on cortical activity. To examine the suggestion, we compared characteristics and effects of SD in the cortex of awake animals under conditions of normal versus slightly enhanced excitability. Hyperexcitable state was produced by pre-treatment with a low dose of pentylenentetrazole (PTZ), an antagonist of gamma-aminobutyric acid (GABA) receptors. GABA is a major driver of neuronal inhibition preventing excessive excitation of the cortex. In patients with frequent (five per month) attacks of migraine with aura, reduced GABA levels in the occipital cortex have been reported [14]. Our study shows that mild cortical disinhibition does not affect properties of cortical SD but strikingly changes its effects on cortical activity in a region-dependent manner.

## 2. Materials and Methods

### 2.1. Subjects

Male Wistar rats (320–400 g, Scientific center for Biomedical Technologies of the Federal Medical and Biological Agency, Russia) were housed in temperature-controlled conditions (22 °C ± 2 °C) on a 12 h/12 h light/dark cycle with food and water available ad libitum. Experiments were designed and performed in accordance with the ARRIVE guidelines and Directive 2010/63/EU for animal experiments. The study protocol was approved by the Ethics Committee of the IHNA RAS (protocol N1 from 1 February 2022). Every effort was made to minimize animal suffering and to ensure reliability of the results.

### 2.2. Stereotaxic Surgery

Under isoflurane anesthesia, electrodes for SD/ECoG recording and guide cannulas for SD induction were bilaterally implanted (10 rats). Recording electrodes (insulated silver wire) were positioned in the frontal (AP: +1.2, ML: ±2.3, DV: −1.8 mm) and occipital (AP: −5.88, ML: ±3.5, DV: −1.5 mm) cortices [15]. A reference electrode was placed over the cerebellum. Stainless steel guide cannulas (23 gauge) were implanted in the somatosensory cortex (AP: −2.8, ML: −4.5; DV: −1.5 mm) of the two hemispheres. The guide cannulas, recording electrodes and pin connector were fixed on the skull bone with acrylic dental plastic. A 30-gauge stylus of the same length as the guide cannula was inserted into it to prevent clogging. Experiments started two weeks after the surgery. During the 3–4 days before the start of experiments, all animals were pre-handled and habituated to the stylus removal.

### 2.3. Initiation of SD and Recording of Cortical Activity

Cortical SD was induced by a pinprick of the somatosensory cortex as described previously [16,17]. Briefly, the needle was inserted into the guide cannula and protruded from its tip by 1.0 mm. The pinprick produces a local microdamage of the cortical tissue, the volume of which does not exceed 0.3 mm^3^ (Figure S1) [17]. Repetition of mechanical stimulation with a weekly interval induces no significant neurodegeneration and neuroinflammation [17]. In each of the 10 rats with the implanted electrodes and cannulas, four tests with induction of unilateral SD were performed with a weekly interval. In tests 1, 2 and 4, cortical SD was induced under drug-free conditions. In test 3, before SD initiation, rats were pretreated with a systemic administration of either saline (control group, *n* = 4) or a low dose (30 mg/kg, i.p.) of GABA(A) receptor antagonist pentylenetetrazol (PTZ, Sigma-Aldrich, St. Louis, MO, USA; experimental group, *n* = 6). In each test, rats were individually placed in a shielded chamber and the implanted connector was attached to the recording cable. Spontaneous cortical activity (ECoG) was recorded before (baseline) and immediately after initiation of a single SD in the cortex of one hemisphere. Fifteen minutes later, a single cortical SD was initiated in the opposite hemisphere. Thus, two unilateral SDs were induced in each test (one SD per side).

Full-band cortical activity (0–100 Hz, 1 kHz sampling rate) was recorded using a four-channel, high-input impedance (1 gΩ) dc amplifier and a/d converter (E14-440, L-Card, Moscow, Russia) with simultaneous video monitoring of behavior. SD was identified by a characteristic high-amplitude negative DC potential shift (the electrophysiological marker of SD). Latency of SD was defined as the period between a pinprick and the onset of DC shift at the recording electrode. Duration of SD was measured from the onset of DC deflection to its return to 90% baseline level.

### 2.4. Data Processing

All signals were checked for artifacts. First, any epoch containing amplitude variation exceeding ±5 absolute deviations from the median of the signal was excluded. Second, segments exhibiting flatlining, defined as periods where the signal derivative remained zero for more than 10 consecutive sampling points, were discarded. Third, any segment displaying a waveform artifact occurring simultaneously on all of the recording channels was rejected as a global, system-wide artifact.

Fourteen artifact-free recordings of SD (seven in control and seven in PTZ groups) were used for assessment of SD characteristics and its effect on cortical activity. Spectral analysis was performed on 600 s, artifact-free segments of ECoG recordings obtained during the baseline period and after the induction of unilateral SD under drug-free conditions (test 2) and after partial disinhibition (PTZ, test 3) in the same rats of the experimental group (*n* = 7). The segments were filtered using Butterworth digital filters: a high-pass filter with a 1 Hz cutoff and a band-stop filter with cutoffs at 48 Hz and 52 Hz. The 600 s epochs were then divided into non-overlapping 20 s intervals and time-dependent changes in mean power for each frequency band were evaluated as described previously [18]. Spectral power was computed using a Fast Fourier Transform (FFT) routine for five frequency bands: delta (1–4 Hz), theta (4–8 Hz), alpha (8–12 Hz), beta (12–25 Hz), and gamma (25–50 Hz). The mean absolute power per interval was calculated for each frequency band. For baseline activity, the mean power was averaged over the entire period of ECoG recording.

### 2.5. Histology

To verify localization of recording electrodes and SD initiation sites, after the end of the experiments, animals were euthanized and perfused intracardially with 0.9% saline. The brains were removed, stored in 10% formalin for 48 h, sectioned in coronal 50 μm slices and stained with 0.1% cresyl violet. A representative brain slice with cannula in the somatosensory cortex is shown in Figure S1.

### 2.6. Statistical Analysis

The analyzed data were distributed normally (Shapiro–Wilk test). Significant difference in spectral power dynamics after SD initiation from baseline level was assessed using one-way ANOVA adjusted for multiple comparisons, with false discovery rate correction using the Benjamini–Hochberg procedure. To compare baseline power under drug-free and PTZ conditions, Student’s *t*-test was used. The latency and duration of repeated SDs were compared using ANOVA for repeated measures, followed by Tukey’s HSD post hoc test. The data were expressed as mean ± SEM with the significance threshold set at *p* < 0.05.

### 2.7. Software and Algorithms

The data analysis was conducted utilizing custom-developed scripts in Python 3.14.2, incorporating the following scientific libraries: Matplotlib 3.10 [19], NumPy 2.3.5 [20] and SciPy 1.16.3 [21].

## 3. Results

**Figure 1:**
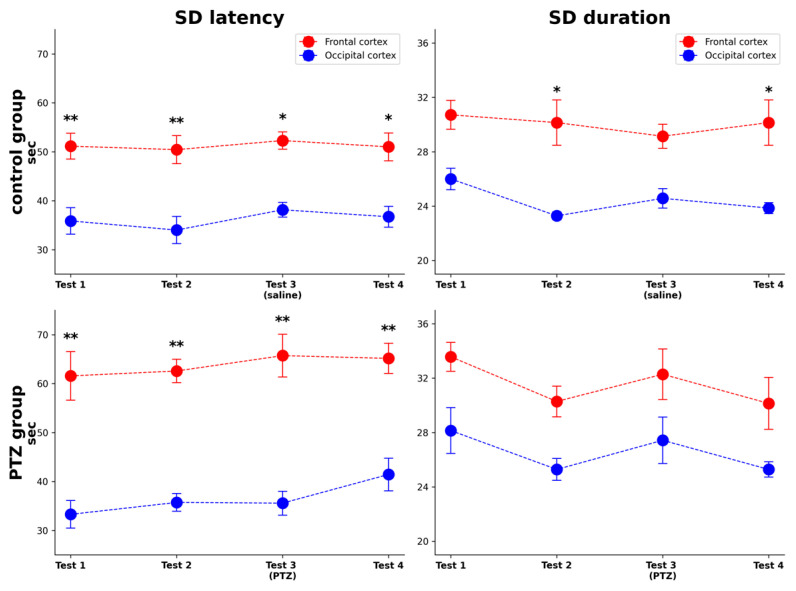
Latency and duration of repeated cortical SDs induced with a weekly interval in awake rats. In each rat from control (above) and experimental (PTZ, below) groups, four SDs were induced in tests 1–4 (a single SD per test) in the somatosensory cortex and recorded in the frontal and occipital cortices. In test 3, rats were pre-treated with saline (control group) or a low dose of pentyleneterazole (PTZ group). Data are presented as mean ± S.E.M. Abscissa: test number (drug-free tests 1, 2, 4; test 3 after saline or PTZ pre-treatment); ordinate: SD latency or SD duration in sec. ANOVA for repeated measures showed no effect of test number on SD latency (F = 0.21, *p* = 0.887 for the frontal cortex; F = 1.712, *p* = 0.182 for the occipital cortex) or duration (F = 1.874, *p* = 0.151 for the frontal cortex; F = 0.281, *p* = 0.839 for the occipital cortex), but showed significant effect of cortical region in control (F = 15.24; *p* = 0.002) and experimental (F = 9.871, *p* = 0.009) groups. ** *p* < 0.005; * *p* < 0.05 by ANOVA for repeated measures with Tukey’s post hoc test.

First, we assessed temporal characteristics of repeated cortical SDs induced with a weekly interval over a month in awake rats. SD was initiated by a pinprick of the somatosensory cortex and recorded in the frontal and occipital cortices. Figure 1 shows the data on the latency and duration of cortical SDs recorded in four repeated tests in rats of control and experimental (PTZ) groups. In both cortical regions, the characteristics of repeated SDs that occurred once a week remained stable across repeated tests. No significant effects of test number on the latency and duration of SD were found. The time of SD propagation to the frontal cortex was longer than that to the occipital cortex (F = 30.38, *p* < 0.001 for control group; F = 64.37, *p* < 0.001 for PTZ group) due to different distances between the site of SD initiation and recording electrodes—4.8 mm in the frontal cortex and 2.8 mm in the occipital cortex. Based on the data, we found that the velocity of SD propagation over the cortex of awake rats was 4.2–4.8 mm/min, which was consistent with our previous data [17]. A significant effect of cortical region on the duration of SD (DC shift) was found in both control and experimental groups with the frontal cortex showing longer duration of SD (Figure 1).

**Figure 2:**
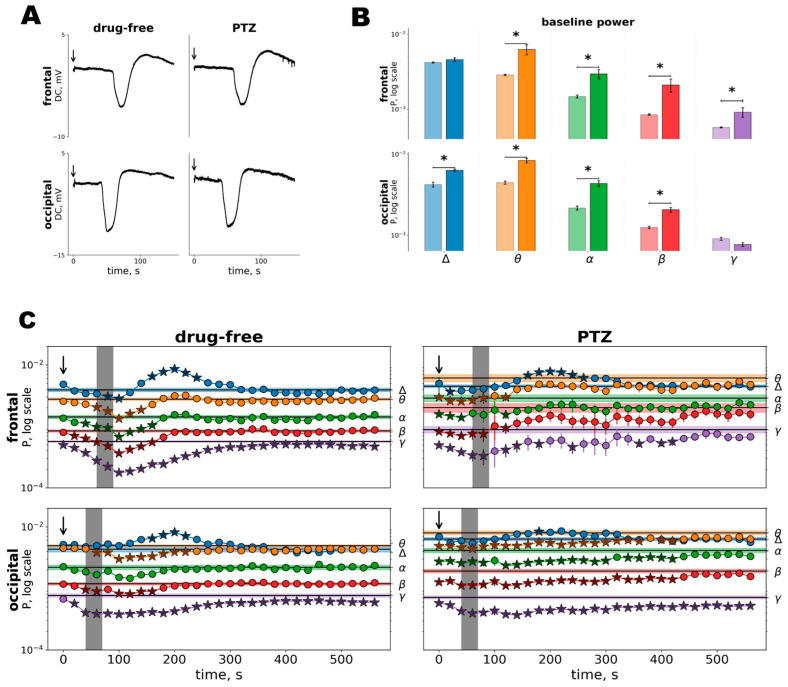
Cortical SD and its effects on electrical activity of the frontal and occipital regions of the cortex with normal and slightly increased excitability. (**A**) The representative recordings of SD in the frontal and occipital cortical regions with normal (drug-free) and enhanced (PTZ) excitability in the same rat. The arrows above the traces mark the timing of the pinprick which initiated cortical SD in each test. (**B**) The mean power of baseline cortical oscillations in drug-free conditions (light colors) and after PTZ pre-treatment (dark colors). Abscissa: frequency bands: Δ (1–4 Hz), θ (4–8 Hz), α (8–12 Hz), β (12–25 Hz) and γ (25–50 Hz); ordinate: log of power. Data are presented as mean ± SEM. * Significant difference between drug-free and PTZ tests (*p* < 0.05, *t*-test). (**C**) The effects of SD on the power of spontaneous oscillations in the frontal (above) and occipital (below) cortices with normal (drug-free) and enhanced (PTZ) excitability. Horizontal lines mark the power of baseline activity in each frequency band. Gray vertical areas in each fragment mark the duration of SD (its depolarization phase). The time of SD initiation is marked by arrows. Stars mark time points at which cortical activity power significantly differed from the baseline level (*p* < 0.05 by one-way ANOVA with Benjamini–Hochberg FDR correction for multiple comparisons).

In test 3, rats were pre-treated with saline (control group) or low-dose PTZ, an antagonist of GABA(A) receptors, to produce mild cortical disinhibition (PTZ group). Administration of PTZ was followed by rapid appearance of spiking and spike-wave activity in all cortical regions that indicated growing cortical excitability (Figure S2). The dose of PTZ neither induced spontaneous SDs nor changed characteristics of cortical SD compared to drug-free tests (Figure 1 and Figure 2A). Although hyperexcitability did not modify depolarization wave (SD), spectral analysis revealed that effects of SD on cortical activity significantly changed.

In the hyperexcitable cortex, the power of baseline cortical oscillations increased compared to drug-free conditions, in the theta, alpha and beta frequency bands in both cortical regions, as well as gamma power in the frontal cortex and delta power in the occipital cortex (Figure 2B). In the drug-free test, SD induced (1) prolonged suppression of gamma oscillations and short-term depression in other frequency bands in the frontal cortex; and (2) long-lasting gamma suppression and mild depression of theta–beta activity in the occipital cortex (Figure 2C). After enhancing cortical excitability (PTZ test), effect of SD on cortical activity dramatically changed: SD-induced depression became minor in the frontal cortex but strengthened in the occipital cortex (Figure 2C). The hyperexcitable occipital cortex showed prolonged depression of spontaneous activity in all frequency bands except delta. Additionally, in PTZ-treated animals, brief wideband depression before the arrival of SD was detected in both cortical areas (Figure 2C). The generalized pre-SD depression developed immediately after the distant initiation of SD in the somatosensory cortex.

## 4. Discussion

The present study shows that slight enhancement of cortical excitability does not change characteristics of SD, but dramatically alters its depressive effect in a region-dependent manner.

We have found that in the hyperexcitable cortex of awake rats, the power of baseline cortical oscillations significantly increases compared to drug-free conditions, especially in the theta–beta frequency bands. Similarly, patients with migraine show a wideband increase in the interictal EEG power compared to healthy controls [22], with the maximal rise in the theta and beta frequency ranges [22,23,24]. The fact that our experimental findings match the clinical data indicates that the low-dose PTZ model is well-suited to study the impact of cortical hyperexcitability in migraine mechanisms.

Our study has shown that for repeated cortical SDs occurring once a week, the pattern of the depolarization phase remains unchanged, even after weak cortical disinhibition. The extracellular negative potential shift during SD reflects simultaneous depolarization of neurons and glial cells in the affected tissue that is produced by the collapse of ion gradients and the rising extracellular potassium level, respectively [2,3,4]. We have found that low-dose PTZ does not affect the duration of SD (depolarization) indicating that susceptibility to SD and the ability to restore membrane potential are similar in the cortex with normal excitability and in the cortex with slightly enhanced excitability. Identical latencies of SD in undrugged and PTZ-treated rats suggest that the reduction in cortical GABA inhibition also does not affect the velocity of SD propagation over the cortex of awake rats. Despite the unchanged depolarization phase of SD in PTZ-treated rats, the depressive effect of SD was significantly altered in the cortex with slightly increased excitability and the pattern of the alterations varied across cortical regions.

In the frontal cortex, mild disinhibition eliminated electrographic depression induced by SD. The same low-dose PTZ abolished SD-induced alterations of functional connectivity without changing the parameters of SD [25]. The findings indicate the involvement of GABAergic transmission in mechanisms of depression induced by SD in the frontal cortex. The suggestion is consistent with the experimental data showing increased inhibitory transmission during neuronal silencing after cortical SD [26,27].

In the occipital cortex, PTZ-induced hyperexcitability produced opposite changes: a prolongation of SD-induced depression. In drug-free conditions, electrographic depression elicited by SD in the occipital cortex was weaker than in the frontal cortex. After weak disinhibition, the SD-induced depression lasted significantly longer in the occipital cortex than in the frontal cortex. Thus, SD can affect ongoing activity of the cortex differently depending on its excitability.

Low GABA levels have been described in the occipital cortex of patients with frequent attacks of migraine with aura [14]. It is suggested that the reduced inhibitory tone in the occipital cortex can make it more susceptible to environmental stimuli, triggering SD. Studies in anesthetized animals confirmed the idea by showing the higher incidence of SD in the occipital cortex compared to other cortical areas [11]. Our present findings provide additional insight into the particular role of the occipital cortex in migraine aura mechanisms. Apart from enhancing susceptibility to SD initiation [11], the hyperexcitable state heightens the sensitivity of the occipital cortex to the depressive action of SD. Our finding that hyperexcitability contributes to the effects of SD on cortical activity can explain why anti-seizure drugs, which reduce brain excitability, are effective in the treatment of migraine [3,4].

Pronounced depression induced by SD in the hyperexcitable occipital cortex but not in the frontal one could underlie a particular role of the occipital cortex in aura mechanisms and the predominance of visual symptoms during migraine aura. Visual aura, the most common in migraine patients, is thought to reflect the propagation of SD over the occipital cortex, and the SD-induced transient neuronal silencing produces negative aura symptoms [3,4,5,6]. A close relation of aura symptoms and suppression of cortical activity has been demonstrated in patients with migraine. Clinical imaging [5], MEG [6] and EEG [7] studies detected unilateral depression of cortical activity and SD-like changes spreading over the hyperexcitable occipital cortex contralateral to visual symptoms during migraine aura.

Another unexpected result of our study is a brief broadband depression of cortical activity that occurs immediately after SD initiation and is detected in both regions of the hyperexcitable cortex. The early short-lasting depression before arrival of SD to the recording sites was never observed in the cortex with normal excitability in drug-free tests. The widespread pre-SD depression recorded in PTZ-treated animals seems to represent an arousal response to mechanical stimulation of the distant cortical region (somatosensory cortex) and suggests an enhanced reactivity of the hyperexcitable cortex to the environmental stress. Previous experimental evidence has shown that increased excitability of the cortex predisposes it to generation of SD by sensory stimulation [28,29].

Potential limitations of the study include a relatively small sampling size and the use of only males. Given the well-known female predominance of migraine, effects of SD in the hyperexcitable cortex of female animals need further investigation.

To sum up, our findings indicate that clinical manifestations of migraine aura depend not only on functional anatomy of cortical regions affected by SD but also on their baseline excitability. The non-uniform depression produced by SD in the cortex with pre-existing hyperexcitability may contribute to the variability in the patterns and intensity of aura symptoms. Also, the data may be important for developing new approaches to the therapy of migraine with aura. If hyperexcitability is a factor contributing to the detrimental effects of SD, pharmacological dampening of cortical excitability can be useful for the alleviation of migraine aura symptoms.

## Data Availability

The data used and analyzed are available from the corresponding author on request.

## Supplementary Materials

The following supporting information can be downloaded at: https://www.mdpi.com/article/10.3390/neurolint18050097/s1, Figure S1: Representative image of the typical lesion produced by the pinprick of the somatosensory cortex. Figure S2: Representative recording of cortical activity after administration of a low dose of PTZ.

## Abbreviations

The following abbreviations are used in this manuscript:

DC	direct current
ECoG	electrocorticography
EEG	electroencephalography
fMRI	functional magnetic resonance imaging
GABA	gamma-aminobutyric acid
MEG	magnetoencephalography
PTZ	pentylenetetrazol
SD	spreading depolarization

## References

[1] Leao A.A.P. (1944). Spreading Depression of Activity in the Cerebral Cortex. J. Neurophysiol..

[2] Somjen G.G. (2001). Mechanisms of Spreading Depression and Hypoxic Spreading Depression-like Depolarization. Physiol. Rev..

[3] Charles A.C., Baca S.M. (2013). Cortical Spreading Depression and Migraine. Nat. Rev. Neurol..

[4] Harriott A.M., Ayata C. (2025). Spreading Depolarization as a Therapeutic Target in Migraine. Nat. Rev. Neurol..

[5] Hadjikhani N., Sanchez Del Rio M., Wu O., Schwartz D., Bakker D., Fischl B., Kwong K.K., Cutrer F.M., Rosen B.R., Tootell R.B.H. (2001). Mechanisms of Migraine Aura Revealed by Functional MRI in Human Visual Cortex. Proc. Natl. Aca. Sci. USA.

[6] Bowyer S.M., Aurora S.K., Moran J.E., Tepley N., Welch K.M.A. (2001). Magnetoencephalographic Fields from Patients with Spontaneous and Induced Migraine Aura. Ann. Neurol..

[7] McLeod G.A., Josephson C.B., Engbers J.D.T., Cooke L.J., Wiebe S. (2025). Mapping the Migraine: Intracranial Recording of Cortical Spreading Depression in Migraine with Aura. Headache.

[8] Aurora S.K., Cao Y., Bowyer S.M., Welch K.M.A. (1999). The Occipital Cortex Is Hyperexcitable in Migraine: Experimental Evidence. Headache.

[9] Ulutas S., Özçelik E.U., Dabó L.G., Bolla F., Tepe N., Yambao P., Ling Y.-H., Pan L.-L.H., Wang S.-J., on behalf of International Headache Academy of the International Headache Society (IHS-iHEAD) (2025). Evoked Potential Studies in Migraine: A Systematic Review of Neurophysiological Patterns across Migraine Subtypes. Cephalalgia.

[10] Gunaydin S., Soysal A., Atay T., Arpaci B. (2006). Motor and Occipital Cortex Excitability in Migraine Patients. Can. J. Neurol. Sci..

[11] Kaufmann D., Theriot J.J., Zyuzin J., Service C.A., Chang J.C., Tang Y.T., Bogdanov V.B., Multon S., Schoenen J., Ju Y.S. (2017). Heterogeneous Incidence and Propagation of Spreading Depolarizations. J. Cereb. Blood Flow Metab..

[12] Vinogradova L.V. (2018). Initiation of Spreading Depression by Synaptic and Network Hyperactivity: Insights into Trigger Mechanisms of Migraine Aura. Cephalalgia.

[13] Smirnova M.P., Pavlova I.V., Vinogradova L.V. (2025). Spreading Depolarization Is Followed by Seizure-like Activation of the Hippocampus: Potential Mechanism of Migraine-Aura Triggered Seizures. J. Headache Pain.

[14] Bridge H., Stagg C.J., Near J., Lau C., Zisner A., Cader M.Z. (2015). Altered Neurochemical Coupling in the Occipital Cortex in Migraine with Visual Aura. Cephalalgia.

[15] Paxinos G., Watson C. (2005). The Rat Brain in Stereotaxic Coordinate.

[16] Samotaeva I.S., Tillmanns N., Van Luijtelaar G., Vinogradova L.V. (2013). Intracortical Microinjections May Cause Spreading Depression and Suppress Absence Seizures. Neuroscience.

[17] Vinogradova L.V., Rysakova M.P., Pavlova I.V. (2020). Small Damage of Brain Parenchyma Reliably Triggers Spreading Depolarization. Neurol. Res..

[18] Medvedeva T.M., Smirnova M.P., Pavlova I.V., Vinogradova L.V. (2024). Different Vulnerability of Fast and Slow Cortical Oscillations to Suppressive Effect of Spreading Depolarization: State-Dependent Features Potentially Relevant to Pathogenesis of Migraine Aura. J. Headache Pain.

[19] Hunter J.D. (2007). Matplotlib: A 2D Graphics Environment. Comput. Sci. Eng..

[20] Harris C.R., Millman K.J., Van Der Walt S.J., Gommers R., Virtanen P., Cournapeau D., Wieser E., Taylor J., Berg S., Smith N.J. (2020). Array Programming with NumPy. Nature.

[21] Virtanen P., Gommers R., Oliphant T.E., Haberland M., Reddy T., Cournapeau D., Burovski E., Peterson P., Weckesser W., Bright J. (2020). SciPy 1.0: Fundamental Algorithms for Scientific Computing in Python. Nat. Methods.

[22] Kim S.J., Yang K., Kim D. (2023). Quantitative Electroencephalography as a Potential Biomarker in Migraine. Brain Behav..

[23] Bjørk M.H., Stovner L.J., Engstrøm M., Stjern M., Hagen K., Sand T. (2009). Interictal Quantitative EEG in Migraine: A Blinded Controlled Study. J. Headache Pain.

[24] Zebhauser P.T., Heitmann H., May E.S., Ploner M. (2024). Resting-State Electroencephalography and Magnetoencephalography in Migraine—A Systematic Review and Meta-Analysis. J. Headache Pain.

[25] Lachinova D.A., Smirnova M.P., Pavlova I.V., Sysoev I.V., Vinogradova L.V. (2024). Transient Destabilization of Interhemispheric Functional Connectivity Induced by Spreading Depolarization. Netw. Neurosci..

[26] Sawant-Pokam P.M., Suryavanshi P., Mendez J.M., Dudek F.E., Brennan K.C. (2016). Mechanisms of Neuronal Silencing After Cortical Spreading Depression. Cereb. Cortex.

[27] Lindquist B.E., Shuttleworth C.W. (2017). Evidence That Adenosine Contributes to Leao’s Spreading Depression in Vivo. J. Cereb. Blood Flow Metab..

[28] Vinogradova L.V. (2015). Comparative Potency of Sensory-Induced Brainstem Activation to Trigger Spreading Depression and Seizures in the Cortex of Awake Rats: Implications for the Pathophysiology of Migraine Aura. Cephalalgia.

[29] Van Harreveld A., Stamm J.S. (1955). Cortical responses to metrazol and sensory stimulation in the rabbit. Electroencephalogr. Clin. Neurophysiol..

