# Supplementary material for: Heightened Sensitivity of the Hyperexcitable Occipital Cortex to Spreading Depression: Evidence for State-Dependent Mechanisms of Migraine Aura
Source: Neurol Int. 2026 May 21;18(5):97. doi: 10.3390/neurolint18050097 (PMC13210180; doi:10.3390/neurolint18050097)
Supplement: Supplementary file 1 [file neurolint-18-00097-s001.zip › neurolint-4254826-supplementary.pdf]

Captions

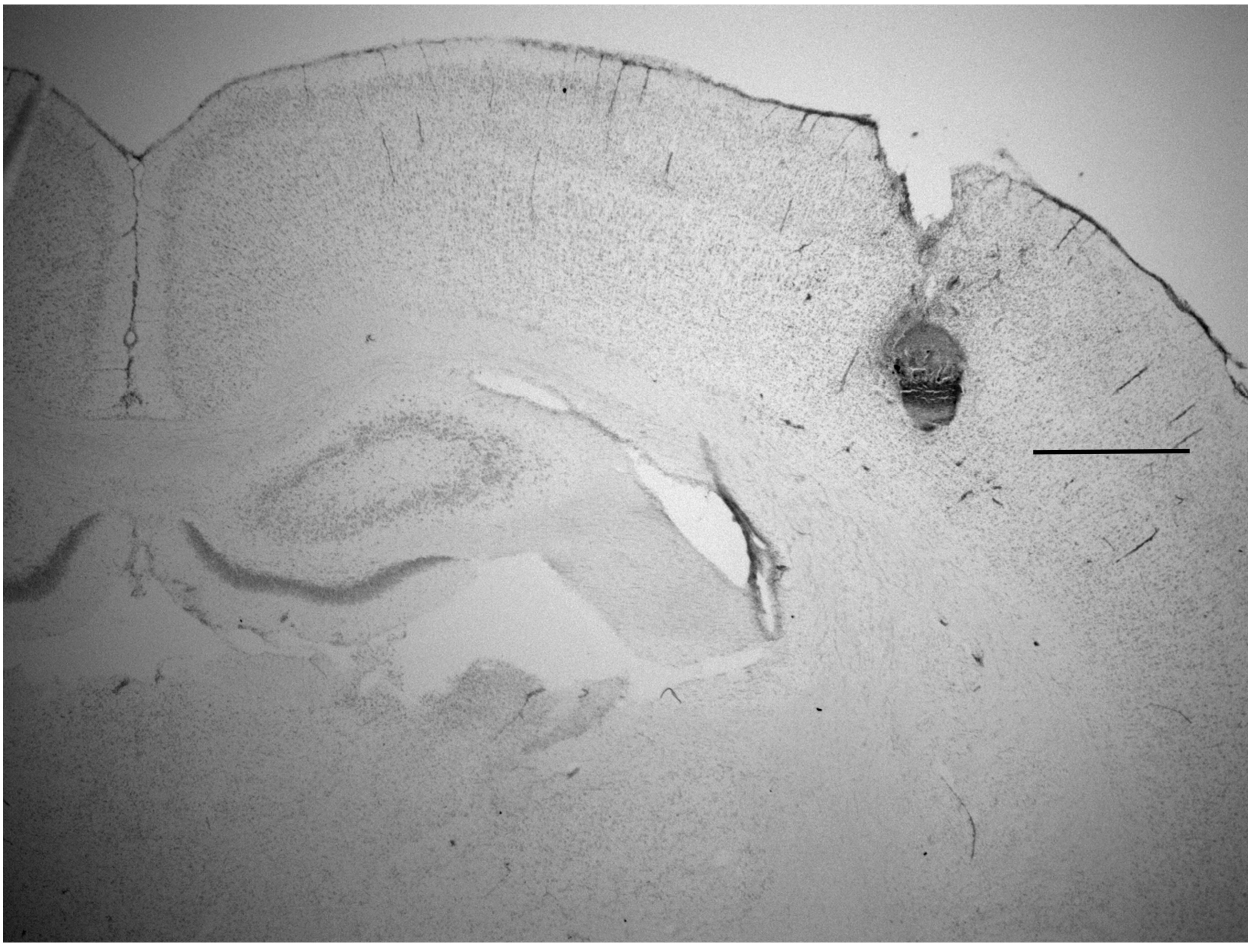

Figure S1. Representative image of the typical lesion produced by the pinprick of the somatosensory cortex. Coronal cortical slice stained by cresyl violet; scale bar is 1 mm.

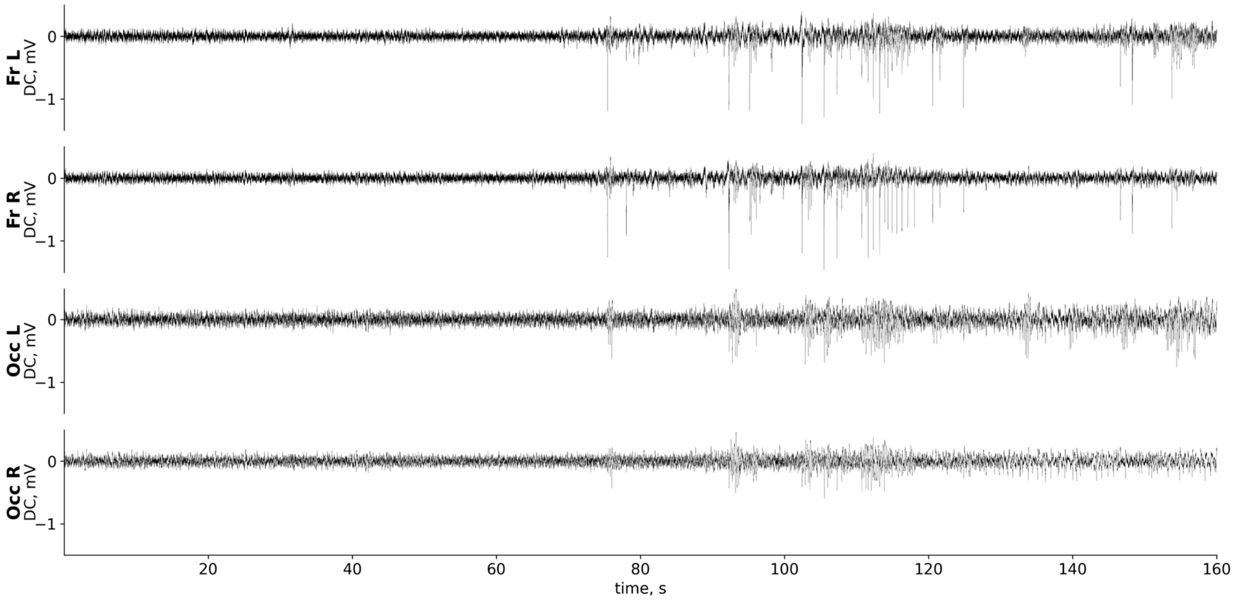

Figure S2. Representative recording of cortical activity after administration of a low dose of PTZ. The PTZ (30.mg/kg, i.p.) was injected immediately before the onset of the recording. The traces show the activity in the left (Fr L) and right (Fr R) frontal cortical regions and in the left (Occ L) and right (Occ R) occipital cortical regions. In about 75 s after PTZ administration, high-amplitude spiking and spike-wave activity appeared in all cortical regions and heralded development of hyperexcitable state of the cortex.
